# Trade policy reform, retail food prices and access to healthy diets worldwide

**DOI:** 10.1016/j.worlddev.2024.106535

**Published:** 2024-05

**Authors:** Rachel Gilbert, Leah Costlow, Julia Matteson, Jakob Rauschendorfer, Ekaterina Krivonos, Steven A. Block, William A. Masters

**Affiliations:** a Friedman School of Nutrition Science and Policy, Tufts University, USA; b Food and Agricultural Organization of the United Nations, USA; c Fletcher School of Law & Diplomacy, Tufts University, 160 Packard Ave., Medford, MA 02155, USA

**Keywords:** Diet costs, Nutrition, Food imports, Import tariffs, Non-tariff measures

## Abstract

Recent use of least-cost diets as a measure of global food security revealed that over 3 billion people are unable to afford sufficient nutritious food for an active and healthy life, driving demand for policy changes to improve access and affordability. This study quantifies the role of imports in consumer prices, matching retail prices in 144 countries to imports by origin of the item or its main ingredient, resulting in a total of 13,846 pairs of a retail price and its import cost in 2017. We find that 55% of retail items had some active imports supplementing domestic production, and of those around 48% have nonzero tariffs whose average effective rate is around 6.7% of the imported commodity price. Over all countries for which data are available, the share of consumer prices for least-cost healthy diets that is attributable to tariffs and non-tariff measures averages 0.67% and 2.45% globally. The highest restrictions are on nutrient-rich vegetables, fruits and animal-sourced foods. Access to bulk commodities from diverse origins is essential for food and nutrition security, providing a greater diversity of foods and food ingredients at lower and more stable prices than can be grown at any one location. On average over all food products that are imported, 83% of the retail price is domestic value added after arrival. We conclude that food imports are best understood as inputs to the domestic production and distribution of retail items, with consumer prices and growth of the food sector dependent on the cost levels, infrastructure and institutions underlying each product’s entire value chain.

## Highlights

• Matching retail food items to traded primary ingredients reveals new stylized facts about food systems and access to healthy diets.
• Most food items worldwide are traded. About 48% of these food items faced tariffs that averaged around 6.7% in 2017.
• Tariffs are highest for vegetables, and highest in low- and middle income countries.
• Taken together, tariffs account for 0.67% ($0.02) of the $3.27 cost per day for a least-cost healthy diet in 144 countries.
• Even in low-income countries, retail food prices are driven mostly by domestic value added after port of entry.

## 1 Introduction

In 2020, a UN flagship report jointly published by FAO, IFAD, UNICEF, WFP and WHO (2020) revealed that about 3 billion people worldwide could not afford a diet facilitative of an active and healthy lifestyle. Based on data from 2017, more recently the same flagship publication updated this figure and concluded that in 2021 more than 3.1 billion people – a staggering 42 percent of the world’s total population – could not afford a healthy diet (FAO, IFAD, UNICEF, WFP and WHO, 2023). These findings, combined with the documented prevalence of various forms of malnutrition in many countries, have sparked strong public interest in policy options to reduce the cost of nutritious foods and improve the affordability of healthy diets.

This paper contributes to policy debates on how best to improve access to healthy diets worldwide. We study the potential of trade policy to improve food access as measured by the affordability of a healthy diet, a statistical concept defined as the least expensive selection of items from a country’s locally available retail items in sufficient quantities to meet requirements for long-term health (Herforth et al., 2020). Using the lowest-cost locally available retail foods to meet national dietary guidelines was introduced as a metric of global food access in 2020, for the flagship annual report on The State of Food Security and Nutrition in the World (FAO, IFAD, UNICEF, WFP, and WHO, 2020), and since 2022 the FAO and World Bank have jointly published annual estimates using the Cost and Affordability of a Healthy Diet (CoAHD) suite of metrics in the widely cited FAOSTAT and World Bank databases (FAO, 2023, World Bank, 2023).

Trade policies can play a key role in economic development with significant impacts on agriculture and food systems and therefore food security and nutrition. Import tariffs have been reduced in the past but remain widely used to protect domestic producers from cross-border competitors, at the expense of making the targeted food and its substitutes more costly for domestic consumers. Linking traded products to retail items, and specifically to consumer prices for the least expensive locally available items used for the new metrics of cost and affordability, is helpful to identify the role of import restrictions in each population’s access to sufficient quantities of nutritious food for an active and healthy life.

The work presented in this paper focuses on two types of trade policy: ordinary import tariffs and non-tariff measures (NTMs). We quantify the contribution of these import measures to retail food prices and the resulting least-cost healthy diet in a large cross-country sample covering 144 economies. Reducing barriers to trade and investment could be especially important for furthering nutritional objectives in low- and middle-income countries: increased trade could help diversify diets with more differentiated retail products that could take advantage of scale economies in agro-processing, eventually lowering consumer prices to expand access to healthy diets.

To quantify how import tariffs and NTMs contribute to the cost of nutritious foods and affect the overall affordability of a least-cost healthy diet, we proceed in two ways that distinguish our work from previous and more traditional research on the effects of trade policy on nutrition and related outcomes. First, we estimate the magnitude of import barriers relative to consumer prices for retail items, in contrast to the traditional focus on wholesale or farmgate prices. This is made possible by converting all quantities from weight or volume to calories, adjusting for variation in moisture content and other factors so as to add up quantities of food needed each day. This approach therefore extends traditional analysis of price transmission and tariff-equivalent policies that focus on cost per kilogram of farm commodities such as Anderson and Masters (2009) and MAFAP (2015), updated to address the role of product transformation before retail sale as in the analysis of value added in food supplies by Yi et al. (2021). Second, we focus on the extent to which import barriers are imposed on the least-cost items in each of the food groups required for health. This approach provides new insights into whether import barriers have a meaningful effect on the economic accessibility of healthy diets, and stands in contrast to the traditional focus on foods used in observed diets, or the starchy staples as an indicator of caloric adequacy.

We find that NTMs are often more costly than import tariffs. NTMs are policy measures other than ordinary customs tariffs that can influence international trade in goods, potentially changing both prices and quantities traded. In the context of food trade, NTMs include Technical Barriers to Trade (TBT), such as labelling or packaging requirements, as well as Sanitary and Phytosanitary (SPS) measures intended to protect human, animal, or plant life or health (UNCTAD & World Bank, 2018, FAO, 2022a). In a global analysis of agricultural trade, Gourdon, Stone and Tongeren (2020) estimate that TBT and SPS measures combined can raise the import prices of agricultural products by almost 15 %. Unlike tariffs, however, NTMs such as SPS rules can play important roles in reducing health risks to consumers (as well as animals and plants), suggesting that the optimal degree of non-tariff trade protection is unlikely to be zero.

These unique analytical components lead to the key insight that trade policy may be a lever of limited potential for reducing the cost of a least-cost healthy diet. Our findings suggest that the share of consumer prices for least-cost retail items attributable to tariffs and NTMs averages 0.6 % and 2.1 %, respectively. These results may be of great value in guiding both trade policy initiatives and investments to improve food access. To the extent that import barriers beyond tariffs and NTMs continue to play a significant role in the cost and affordability of healthy diets, additional trade facilitation measures for raw commodities at the border would be needed to bring healthy diets within reach of all people at all times. Then, beyond the cost of food products at the port of entry, identifying the magnitude and role of domestic costs for product transformation, distribution and retailing reveals the need for innovation and investments to improve access for consumers at the point of sale within each country.

Our study contributes to several strands of the growing body of work investigating the links between trade, trade policy and nutrition. A first body of work our study complements is empirical research on the effects of trade policy on the availability or consumption of foods with different nutritional value. For example, Mendez Lopez et al. (2017) find that higher tariffs on sugar-sweetened beverages significantly decrease per capita imports and reduce sales of these products in lower-middle income countries. Evidence from Fiji suggests that lowering of tariffs levied on nutritious fruits and vegetables led to higher imports of this food group and increased domestic availability of these products (Bell et al., 2020). Closely related, a second strand of literature shows that tariff reductions or higher imports of energy-rich foods are associated with adverse effects on health-related indicators at the individual level, such as prevalence of obesity. These findings hold for countries at different development stages. To illustrate, Miljkovic et al. (2018) find that an increase in trade openness leads to higher overweight and obesity ratios in Brazil. Giuntella, Rieger and Rotunno (2020) find that higher exposure to imports of foods classified as “unhealthy” significantly contributed to the rise of obesity in Mexico. At the global level, Abay, Ibrahim and Breisinger (2022) find that a reduction in tariff rates on sugars, confectionary products, fats, and oils is associated with higher body weight, overweight, and obesity rates, with more pronounced effects among the poor. Adding to this, and focussing their discussion on sub-Saharan Africa, Boysen et al. (2019) find that an increase in the tariff difference between highly processed and less processed foods is negatively associated with obesity and positively with prevalence rates for underweight.

A third body of work considers diet-related impacts of trade agreements rather than focusing solely on import tariffs. For example, Unar-Munguía, Monterubio Flores and Colchero (2019) and Barlow et al., (2017b) examine the effects of the North American Free Trade Agreement (NAFTA) on supply and consumption of sweeteners in Mexico and Canada, respectively. In both cases these studies find that joining NAFTA led to higher (apparent) consumption or supply of sugar and sweeteners. Similarly, Baggio and Chong (2020) find that a country entering an FTA with the US had an increase in obesity prevalence between 4.4 percent and 9.8 percent, compared with a country without such an agreement in place. Schram et al. (2015) find an increase in soft drinks sales in Vietnam after the country became a WTO member. In a review of 17 quantitative studies, (Barlow et al., 2017a) conclude that there was consistent evidence that implementing trade agreements was associated with higher consumption of sugar-sweetened beverages and processed foods. Related work by Law (2019) finds large effects of India’s unilateral trade reform on diet diversity among rural Indians. Fourth, and complementing these empirical studies, ex-ante analysis with Computable General Equilibrium (CGE) models is often used in the literature to assess the potential impacts of eliminating import barriers on food prices and the cost of healthy diets. A recent example includes the analysis using the MIRAGRODEP model to assess the possible effects of eliminating price incentives to producers (i.e. border measures and market price controls), as reported in FAO, IFAD, UNICEF, WFP and WHO (2022). The authors find that removing border support on nutritious products while keeping unchanged support for products of high energy density and minimal nutritional value would result in a reduction in the Cost of a Healthy Diet (CoHD) by 1.7 percent (Glauber and Laborde, 2023). The effects are particularly pronounced for Asia and the group of lower-middle-income countries.

In contrast with the body of work cited above, our study provides new evidence on the potential of trade policy to make healthy diets more affordable, which is a previously unstudied outcome of considerable importance to public policy. In addition, the novel and harmonized and global data set we create – linking trade policy and traded primary ingredients directly to final retail items – provides a valuable resource for further research. For example, future work could build on the data assembled for this study, adding supply and demand responses to trace how public policy changes would alter quantities and lead to new equilibrium prices. Our dataset is also distinctive for its granularity, including estimates for tariff costs at the food item level. This improves upon datasets used for CGE models, which typically rely on estimates for producer support that are only available for major commodity groups and not at the level of individual food items.

## 2 Least-cost healthy diets

The data and methods used to compute least-cost healthy diets provide a price index for retail foods as inputs to a population’s long-term health, in contrast to the consumer price index that reflects foods actually consumed based on ability and willingness to pay for different foods based on their taste, culinary habits, time, and fuel costs for meal preparation, as well as commercial marketing, culture, and aspirations that influence food choice. As shown in the literature review of Masters, Finaret and Block (2022), individuals cannot observe the nutritional composition of food, or know how that composition affects their disease risk and longevity, and consumers also have other objectives in addition to health. Computing least-cost healthy diets reveals whether a population that consumes unhealthy diets does so because of poor access, due to either unusually high prices or insufficient income to buy the least expensive locally available healthy diets, or because healthful items are displaced by unhealthy diets due to the many other attributes of each item that drive food choice.

To measure each population’s access to sufficient foods for an active and healthy life, we use the Cost of a Healthy Diet (CoHD) method introduced for global monitoring jointly by the FAO (2023) and World Bank (2023). The CoHD is defined as the cost per day for the least expensive retail items available to a population, in sufficient quantities of each food group needed for long-term health as specified in food-based dietary guidelines (FBDGs). The specific food group targets used for CoHD form a composite “healthy diet basket” that meets commonalities among national FBDGs, as described in Herforth et al. (2022) and spelled out in Table 1.

**Table 1:** Composition of the global Healthy Diet Basket target intake.

Food Group	Number of food items selected	Total energy content (kcal)	Typical weights of example foods (g)
Starchy staples	2	1160	322 g dry rice
Vegetables	3	110	270–400 g
Fruits	2	160	230–300 g
Animal-source foods	2	300	210 g egg
Legumes, nuts, seeds	1	300	85 g dry bean
Oils and fats	1	300	34 g oil
Total	11	2330	

*Source:* Herforth, A., Venkat, A., Bai, Y., Costlow, L., Holleman, C. & Masters, W. 2022. Methods and options to monitor the cost and affordability of a healthy diet globally: Background paper for The State of Food Security and Nutrition in the World 2022. FAO Agricultural Development Economics Working Papers 22. Rome, Italy, FAO. https://doi.org/10.4060/cc1169en.

The quantities of each food group are specified in calorie terms to allow substitution between items with similar nutritional composition, while maintaining energy balance. Quantities of each food group that add up to 2,330 kcal/day would be sufficient for an active adult woman at the median height and weight of the WHO reference population, which happens to also be the average requirement over all sex-age-year groups age three years and older in the WHO reference population (Herforth et al., 2022, Schneider and Herforth, 2020). Defining a healthy diet basket in this way allows for variation in the volume and water weight of locally available items, while maintaining energy balance and sufficient diversity of food sources within and between groups to meet a population’s health needs as specified in national dietary guidelines.

## 3 Conceptual framework

**Figure 1:**
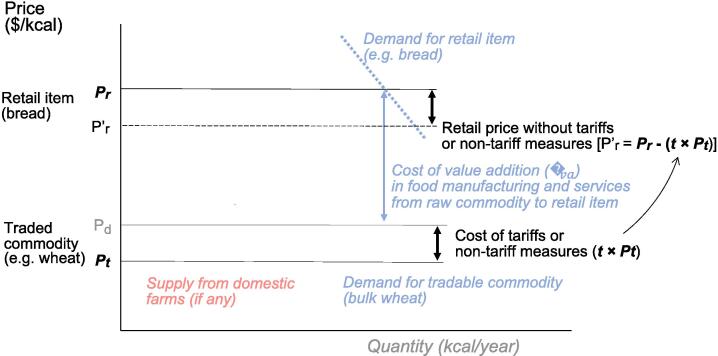
Conceptual model of value added from imports to retail prices.

This study uses data on tariffs and non-tariff measures imposed worldwide to quantify the contribution of import barriers to CoHD. Figure 1 shows our conceptual model of a country’s market for a food product, expressed in US dollars per calorie of its principal ingredient. We illustrate this with the example of imported wheat as the principal ingredient in bread, linking the price paid for imports including cost, insurance and freight of the traded product (*P_t_*), to the domestic cost of buying that traded commodity at its port of entry within the country (*P_d_*) and transforming it through value addition in the food manufacturing and service sector for packaging and transport, storage and distribution to the final price paid by consumers for the corresponding retail item (*P_r_*).

The bulk commodity version of the internationally traded product costs *P_t_* to bring into the port of entry. Costs to clear customs for onward shipment to a distributor involve payment of taxes and compliance with NTMs, both of which are typically reported in ad valorem terms as a proportional tariff-equivalent rate (*t*) charged on the price paid for the traded commodity (*P_t_*). The resulting price per unit of the bulk commodity for domestic buyers is just one input into food manufacturing and distribution. This includes the costs of value addition (*C_va_*), which are held constant in Figure 1 since by definition they consist of things other than the traded product itself.

The variables in this conceptual model for which we have observed estimates are *P_t_* and *P_r_* (in US dollars per unit), and *t* (in proportional ad-valorem terms). From those data we can determine what fraction of *P_r_* is accounted for by those tariffs and non-tariff measures paid on imports of the main ingredient, which is (*t × P_t_*)/*P_r_*.

The conceptual model of food value chains shown in Figure 1 is distinctive in that all prices are converted to US dollars *per calorie* of each product. That data transformation, made possible by matching item descriptions to food composition data for retail products and the corresponding traded commodity, allows us to trace the costs of a retail item back to the principal form in which that product might be imported, even when transformation alters the product and adds or removes water weight. Items are matched only to their principal ingredient. In practice, almost all least-cost items are single-ingredient foods with only water added and inedible portions of the food removed, with additional ingredients that have little effect on the item’s macronutrient content. Accounting for multiple ingredients would require data on the ratio of those ingredients. Such data would be helpful for studies of how trade affects prices for packaged foods like tinned soups or breakfast cereals, but these items do not appear in least-cost diet baskets and are therefore not relevant for this analysis focusing on access to the least expensive items in each food group.

For each imported commodity in each country, the domestic price after paying tariffs and the cost of compliance with NTMs is:

(1) $$P_{d}=\;P_{t}\left(1+t\right)$$

The consumer price paid for the retail form of each product is then:

(2) $$P_{r}=P_{d}+C_{\mathrm{va}}$$

In this formulation, *C_va_* is the cost of value addition, which consists of labour and returns to management, plus rental or capital cost of ownership for facilities and equipment as well as energy costs and other inputs needed to operate each stage of the value chain from the port of entry through transport and storage, transformation, and distribution of the product in retail form. By this definition, *C_va_* includes all costs associated with the retail item other than the cost of its primary ingredient, which may be subject to import restriction. Accordingly, we hold *C_va_* constant, allowing us to calculate what retail prices would be with varying degrees of trade barrier reduction ($P_{r}^{\prime }$), by subtracting a given proportion of trade barrier costs from the baseline retail price:

(3) $${P_{r}}^{\prime }=P_{r}-\left(t{\times P}_{t}\right)$$

In equation (3), prices, tariffs, and NTMs are measured as USD per calorie, with tariffs and NTMs converted from ad valorem equivalents to their specific cost. Equivalently, we can write equation (3) as:

(3′) $$P_{r}^{\prime }-P_{r}=-\left(t{\times P}_{t}\right)$$

and in proportional terms, the difference in retail prices depends on the traded commodity’s cost share of retail prices:

(3″) $$\frac{P_{r}^{\prime }-P_{r}}{P_{r}}=-t\times \frac{P_{t}}{P_{r}}$$

We calculate the magnitude of tariffs separately from NTMs, first because those are distinct policy measures and also because we have different numbers of countries with available data for tariffs and NTMs.

Finally, we use each set of retail prices to identify least-cost healthy diets in each country, adding up prices of the least-cost items within each food group in the proportions needed for health. In each scenario, the resulting CoHD in each country is a weighted sum of least-cost items in the healthy diet basket, as follows:

(4) $$\mathrm{CoHD}=\sum_{i=1}^{n}min\left(P_{\mathrm{ir}}q_{i}\right)$$

where the quantities required for each item ($q_{i}$) are as specified in Table 1, and the particular items selected for CoHD are those with the lowest cost per calorie for that food group. Recalculating CoHD allows for the selection of a different basket when the reduction in trade barrier costs results in new least-cost items, allowing us to calculate the proportion of each country’s diet costs that is attributable to payment of import tariffs or compliance with non-tariff measures as $(CoHD-CoHD^{\prime })/CoHD$.

In calculating the relative importance of tariffs and NTMs worldwide in determining access to a least-cost healthy diet we hold all else constant, including each partner’s share of imports and trade unit values. Our focus is purely on prices in the base year of 2017, estimating the share of retail item prices that is the cost of bilateral tariff payments or compliance with non-tariff measures. Future efforts to estimate quantity changes under specific policy scenarios might account not only for supply and demand response but also for substitution among sources of imports. The new equilibrium in such a model would depend on model structure and parameters, including the degree to which resources can be reallocated in response to price changes.

## 4 Data sources

The starting point for our analysis is the availability and price of retail food items in each country, as reported by national governments to the ICP to compare price levels for computing purchasing power parity exchange rates. Each observation is a single national average price for a standardized item considered widely available in that country. In 2017, the ICP provided retail prices for 680 foods and non-alcoholic beverages across 177 economies. We convert retail prices into 2017 PPP per kilocalorie of edible matter using food composition data from the United States Department of Agriculture (USDA) and the West African Food Composition Table, and the ICP 2017 PPP data (US Department of Agriculture, Agricultural Research Service., 2016, Vicent et al., 2020).

We match ICP retail food and beverage items to the primary FAO traded food commodity corresponding to the same food product, using detailed metadata provided by the ICP and product category descriptions from FAO for the FAO Commodity List (FCL). In some cases, traded commodities and retail items do not align with a one-to-one concordance. For a subset of items for which multiple matches are both possible and plausible, we use trade flow data to match each ICP retail item to the FAO traded food commodity with the largest value of imports. The full details of our data collection and processing procedures are available in the online appendix to this paper.

We use tariff and NTM data from the UNCTAD TRAINS database. The bilateral tariff data are published as ad valorem equivalents (AVEs) at the 6-digit Harmonised System (HS) level through the WITS platform hosted by the World Bank. To apply the appropriate tariff and NTMs to each traded food commodity, we match ICP retail food items-FAO traded food commodity pairs (ICP-FCL pair) to the appropriate 6-digit harmonized system code. Where multiple possible HS6 codes can be matched to a single ICP-FCL pair, we choose the HS6 code corresponding to the least-processed product group.

In light of the ambiguous correspondence between FCL and HS6 codes, and following Headey, Hirvonen, and Alderman (2023), we perform a separate sensitivity analysis to estimate the range of tariff costs to which each FCL commodity may be subjected. We match each FCL commodity to all possible HS6 codes in the FAOSTAT concordance, then merge with tariff AVEs from UNCTAD. We keep two HS6 matches for each FCL commodity: one with the lowest AVE and one with the highest AVE. We then calculate the contribution of tariffs to CoHD using this dataset, separately estimating an upper-bound case where each item is subjected to the lowest possible tariff and a lower-bound case where each item is subjected to the highest possible tariff. We do not perform a sensitivity analysis for NTMs, since for many FCL commodities all potential HS6 matches are within the same GTAP sector and thus would be subject to trade costs of the same magnitude within our analytical model.

To assess the contribution of NTMs to the cost of healthy diets, we combine a binary indicator of whether NTMs are imposed on a given product from a given trading partner with estimates of the ad-valorem equivalents of NTMs. The AVEs are price-based estimates averaged across all countries and grouped by GTAP sector for four categories of NTMs (Cadot et al., 2018).

Descriptive statistics of the data that drive our results are provided in Table 2, showing the fraction of all retail items with consumer prices that are matched to a traded commodity, the share of the unit value of the imported primary ingredient in the final product’s retail price, the fraction of those items that are subject to nonzero import tariffs, and the magnitude of those tariffs. The combined dataset includes 13,846 country-retail food observations across 144 countries, of which 48% are potentially subject to tariffs.

**Table 2:** Extent to which retail foods and least-cost foods are traded and tariff-laden.

	**All items**					**Least-cost items**				
	**Pct of items matched to an imported commodity unit value**	**Unit value of imported commodity as % of retail price**	**Pct of imported commodities with tariffs**	**Ave. tariff, where present**	**N**	**Pct of items matched to an imported commodity unit value**	**Unit value of imported commodity as % of retail price**	**Pct of imported commodities with tariffs**	**Ave. tariff, where present**	**N**
	**(a)**	**(b)**	**(c)**	**(d)**	**(e)**	**(a)**	**(b)**	**(c)**	**(d)**	**(e)**
Starchy staples	71 %	0.10	64 %	6.03	3,473	68 %	0.18	62 %	3.51	288
Vegetables	56 %	0.20	52 %	6.70	1,420	59 %	0.24	56 %	7.97	432
Fruits	63 %	0.16	58 %	6.37	1,482	55 %	0.19	51 %	4.11	288
ASF	37 %	0.27	29 %	7.57	5,699	30 %	0.35	25 %	4.03	288
LNS	75 %	0.13	69 %	6.03	826	78 %	0.17	74 %	4.06	144
Oils and fats	72 %	0.17	63 %	7.66	946	57 %	0.22	51 %	4.65	144
*Total: all foods*	55 %	0.17	48 %	6.68	13,846	56 %	0.22	52 %	5.15	1,584

***Notes***: Data shown are for all retail items on the left, and only items selected in least-cost diets on the right. Column details are (a) Percentage of all country-retail food combinations for which the retail item was successfully matched to a primary ingredient from the FAO Commodity List, where that country is a net importer of the commodity and the commodity has a non-missing unit value; (b) percentage of the final product’s retail price that is accounted for by the trade unit value of the corresponding imported commodity; (c) percentage of those imported commodities that have any tariffs; (d) average tariff for all items in column c; (e) total country-retail food item observations. Food group abbreviations are ASF = animal-source foods; LNS = Legumes, nuts, seeds.

Table 2 reveals that most retail items could be matched to an imported commodity with a known unit value, especially for starchy staples and legumes, nuts and seeds, for which over 70% of items are importable in this sense. The retail items that are least often matched to an importable commodity are animal source foods, whose products are imported for only 37% of all retail items and 30% of the least-cost animal source items. This finding is limited by our analytical approach, which does not account for increased trade after tariff liberalization. Eliminating high tariffs could lead to substitution of domestically goods with cheaper imported equivalents, which could then increase the selection frequency of imported items in least-cost diets. In such cases, the estimates presented here of the contribution of tariffs to diet costs are biased toward the null.

The cost (trade unit value) of the primary imported commodity ingredient accounts for 17% of retail prices for all items, and 22% of retail prices for least-cost items. In addition to that cost, tariffs are imposed on 48% of all commodities and 52% of least-cost items, and those tariffs that are imposed average around 6.7% and 5.2%, respectively. Most of the retail cost is value added beyond the tariff-laden price of imported products, with some interesting differences between food groups and for least-cost items. For all food groups except vegetables, least-cost items are subject to smaller tariffs than more expensive items in that food group. Relatively high protection to domestic producers of vegetables is consistent with explanations of trade policy based on the relative power of interest groups, as described for example in Anderson et al. (2013). In these explanations, observed policies tend to be actions such as vegetable import tariffs whose costs are dispersed as a small price rise for the entire population of consumers, while delivering visible and concentrated benefits to specific producers.

## 5 Results

Trade barriers on imported commodities account for a small but measurable fraction of retail prices for least-cost healthy diets globally, as shown in Table 3. We estimate that tariff payments account for 0.67% of healthy diet costs in the 144 countries for which we have data. Compliance with NTMs accounts for a larger portion of diet costs at 2.45% worldwide. Table A1 (see online appendix) highlights results for three illustrative countries. We also find that the same items remain the least expensive option within each food group.

**Table 3:** Percentage of the Cost of a Healthy Diet attributable to trade barriers.

	**%**
**Cost of tariffs (percent of import price)**	
*By food group*	
Starchy staples	0.06
Vegetables	0.26
Fruits	0.12
Animal-source foods	0.16
Legumes, nuts, seeds	0.06
Oils and fats	0.02
**Total over all items in a healthy diet**	**0.67**
*Number of countries*	*144*
**Cost of non-tariff measures (pct of import price)**	
**Total over all items in a healthy diet**	**2.45**
*Number of countries*	*105*

***Note***: Data shown are average percentages of each country’s total cost per day for a healthy diet basket.

The magnitude of tariff and NTM costs as a share of retail diet costs shown in Table 3 is driven by the descriptive statistics shown in Table 2: focusing on the set of all least-cost items in our dataset, more than half (56%) are matched to imports while a slightly smaller fraction (52%) is subject to tariffs. Where tariffs are in place, the average applied rate is relatively small (5.15%), and those tariffs are applied to imported commodities whose value is a small share of retail product prices (22%). For most foods in least-cost diets, most of the retail cost is accounted for by labour, facilities and other value added in transforming, distributing, and selling the final product. The workers and companies that generate this value added rely on having reliable access to imported commodities, but the value added itself reflects domestic investments and labour costs that are not traded internationally.

These results are robust to a sensitivity analysis presented in Table A2 (see appendix), which illustrates the range of potential tariff costs as a fraction of CoHD for each country in the dataset, with variation stemming from the imperfect match between imported FCL commodities and HS6 tariff codes. Standard deviations are high for several countries where ambiguity in tariff application was most consequential, particularly Morocco, Cambodia, and Kenya, as well as several other African and Caribbean countries. However, 126 out of 144 countries had standard deviations smaller than 1, indicating a higher degree of certainty around our estimates of tariff costs. For most countries, the difference in upper and lower bound tariff costs associated with matching to different HS6 codes is extremely small, not exceeding 9 cents for countries with standard deviations less than or equal to 1.

**Figure 2:**
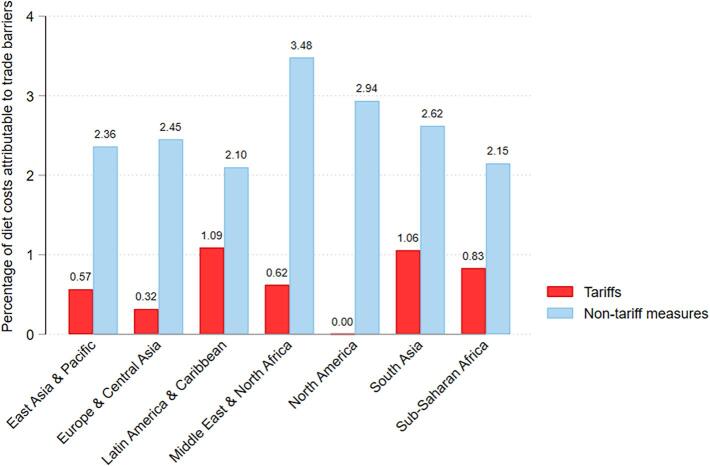
Percentage of diet costs attributable to trade barriers by region. *Notes*: Data shown are the global mean cost per day. Data on NTMs are only available for N = 105 countries.

**Figure 3:**
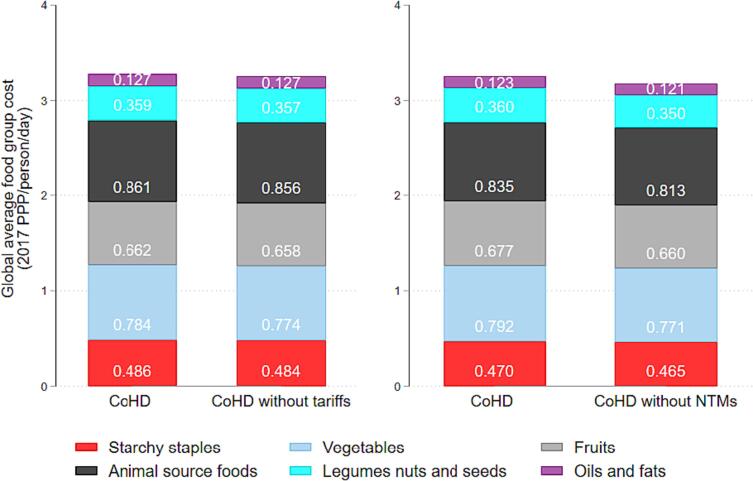
Global average food group cost, with and without trade barriers. *Notes*: Results from North America are excluded due to very low country sample size (n = 2).

Figure 2 demonstrates substantial variation in the contribution of tariffs and NTMs to least-cost diets across regions. At the regional level, tariff costs contribute the most to CoHD in Latin America and the Caribbean and South Asia. Figure 3 disaggregates these results by food group at the global level, illustrating the cost per day of each HDB food group, both with and without tariffs and NTMs. The cost of consuming enough of the least-cost animal-source foods is the most affected by tariff measures. Globally, the average least-cost way to meet animal-source foods (ASF) needs per person per day is USD 0.861 when trade barriers are in place, compared to 0.856 without tariffs. Among the 105 countries for which NTM data are available, NTMs on ASFs and vegetables contribute most to the cost of healthy diets.

Disaggregating across both regions and food groups, Table 4 demonstrates the substantial heterogeneity underlying the previous aggregates. At the global level, the largest trade barrier costs derive from import measures applied to vegetables and animal-source foods. Barriers on animal-source foods have the largest impact on least-cost diets in South Asia, Middle East and North Africa (MENA), and Sub-Saharan Africa (SSA). The vegetable food group is subject to high trade barrier costs in all regions except for MENA and North America, with particularly large costs in Latin America and the Caribbean (LAC). Indeed, least-cost vegetables face higher tariffs when compared both to vegetables in general and to other least-cost foods, with tariffs averaging 6.7% for all items and nearly 8% for all least-cost foods.

**Table 4:** Share of diet costs attributable to import barriers, by region and food group.

	**EAP**	**ECA**	**LAC**	**MENA**	**NA**	**SA**	**SSA**	**Global**
	**%**	**%**	**%**	**%**	**%**	**%**	**%**	**%**
**Tariffs**								
*By food group*								
Starchy staples	0.11	0.01	0.14	0.02	0.00	0.08	0.07	0.06
Vegetables	0.24	0.14	0.60	0.04	0.00	0.31	0.25	0.26
Fruits	0.11	0.12	0.05	0.04	0.00	0.09	0.21	0.12
Animal-source foods	0.06	0.02	0.17	0.39	0.00	0.28	0.25	0.16
Legumes, nuts, seeds	0.02	0.02	0.12	0.10	0.00	0.21	0.03	0.06
Oils and fats	0.02	0.01	0.02	0.03	0.00	0.09	0.02	0.02
**Total over all items in a healthy diet**	0.57	0.32	1.09	0.62	0.00	1.06	0.83	0.67
*Number of countries*	*16*	*43*	*26*	*15*	*2*	*7*	*35*	*144*
**Non-tariff measures**								
**Total over all items in a healthy diet**	2.36	2.45	2.10	3.48	2.94	2.62	2.15	2.45
*Number of countries*	*13*	*34*	*22*	*12*	*2*	*5*	*17*	*105*

**Figure 4:**
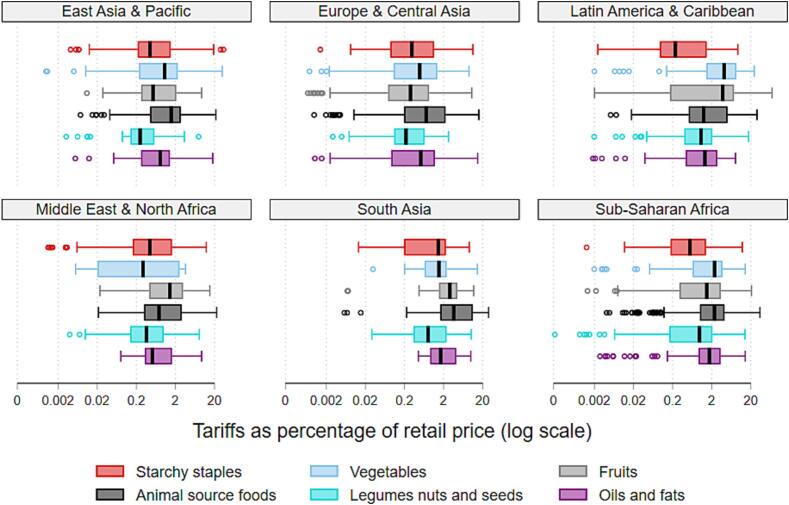
Share of retail prices attributable to import barriers, by region and food group.

Figure 4 expands on Table 4, providing greater insight into the dispersion of tariff burdens around the median for each food group by region. For example, the distribution of tariff burdens on fruits in LAC is strongly left-skewed suggesting greater concentration across countries at the high end of the distribution. Most of the outliers in our results fall on the low end of the distribution.

There is also substantial national variation within each region as illustrated in Figure 5a, Figure 5b. Tariffs contribute disproportionately to the cost of least-cost diets in several countries in Western Africa, Eastern Africa, South and Southeast Asia, and several Central American and Caribbean nations. In Ethiopia and Ghana, tariff costs exceed 4 percent of CoHD, while costs exceed 3 percent of CoHD in St. Lucia, Antigua and Barbuda, St. Vincent and the Grenadines, Fiji, and Panama. Tariff costs range from about 2.5 to 3 percent in Belize, Bangladesh, Mali, Israel, Algeria, Korea, and Nigeria.

**Figure 5a:**
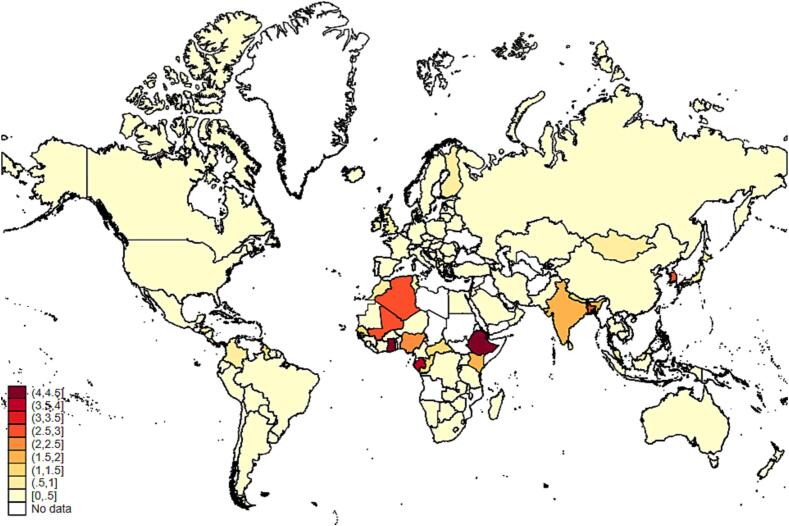
Contribution of tariffs to diet costs by country (% of CoHD).

**Figure 5b:**
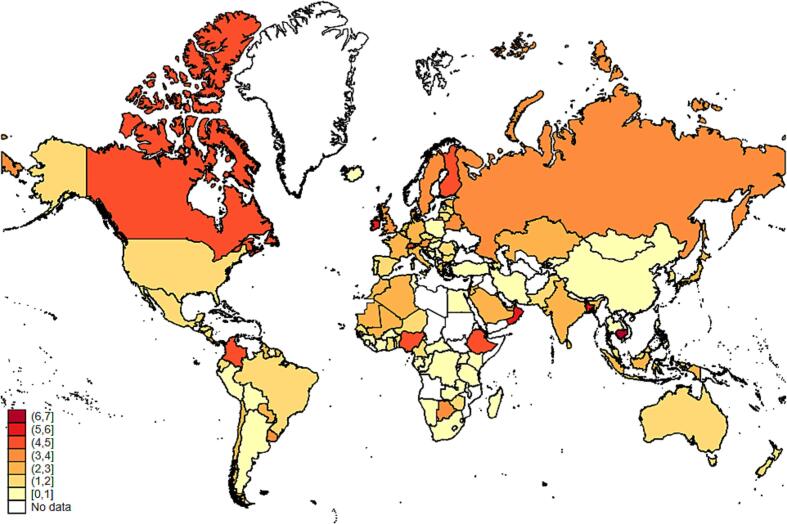
Contribution of NTMs to diet costs by country (% of CoHD).

This distribution of elevated trade costs persists in the sensitivity analysis presented in Table A2. When a wider range of possible tariffs is included in the analysis, tariff costs become much higher for Morocco (5.3%), and Cambodia (3.11%), but average tariff costs shift only marginally for other countries.

There is less national variation in NTM costs, as NTMs contribute more substantially to diet costs in countries across all regions. Variation may also be due to incomplete reporting of NTMs. However, some countries are regional outliers with respect to NTM costs, including Bangladesh (5% of CoHD compared to a regional median of 1.8 for South Asia), Singapore and Cambodia (6% compared to 1.6% for East Asia and Pacific), Israel, Oman, Bahrain, and Kuwait (5 to 6 percent compared to 2.4 for MENA), and Canada (4.1% compared to 1.7 for the United States).

**Figure 6:**
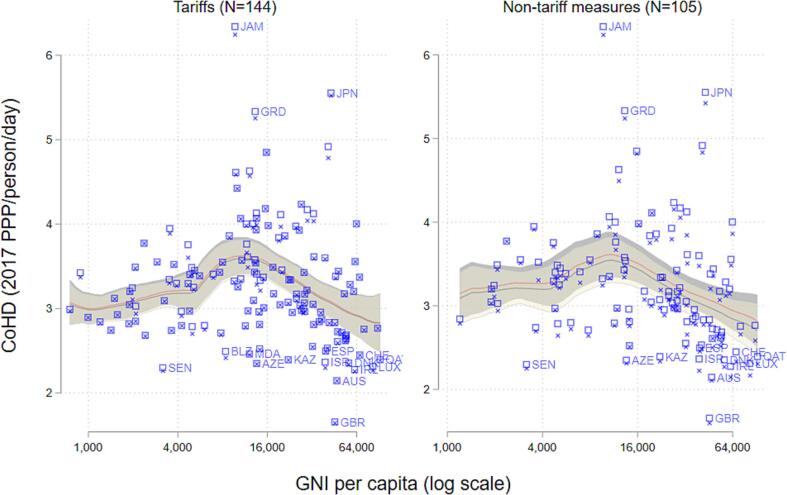
Cost of a Healthy Diet with and without trade barriers.

National income per capita has a distinct correlation with CoHD both before and after subtracting the cost of compliance with tariffs and NTMs. Figure 6 shows the level of CoHD for all individual countries in our sample with and without tariffs (left panel) and non-tariff measures (right panel). A non-parametric line shows the mean and 95% confidence interval for diet costs at each income level, at observed prices (red) and without trade barriers (gray). Diet costs are moderately high in low-income countries and highest in middle-income countries. As national income continues to rise, this trend reverses and CoHD declines such that CoHD is lowest in the wealthiest countries. Bai et al. (2021) explain a similar cross-country pattern in diet costs as partially resulting from greater electrification and denser marketing chains in wealthier countries, which disproportionately reduce the costs of perishable foods.

These results also reveal countries where small differences in diet costs arise due to trade barriers. Each country’s diet costs are plotted as both an X (for diet costs net of trade barriers) and a square (for diet costs at observed retail prices). The vertical distance between these two observations indicates the cost of compliance with trade barriers. Tariffs only result in an appreciable gap between diet costs for a handful of countries, while non-tariff measures have a larger and more consistent effect across countries and national income level.

**Figure 7:**
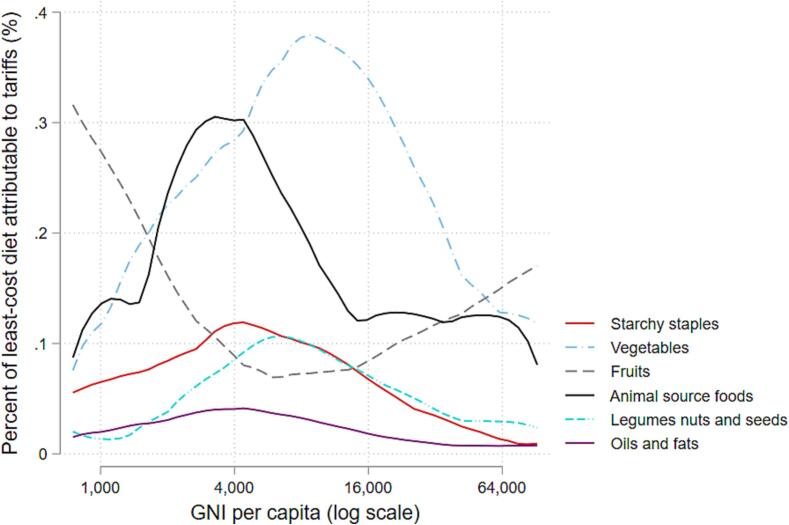
Percent of least-cost diet attributable to tariffs, by food group.

Figure 7 extracts just the non-parametric line to show differences in the percentage contributions of trade barriers to least-cost healthy diets by food group at each level of national income per capita. Here we find wide variation across food groups. Tariffs on least-cost vegetables and ASFs make the largest contribution to diet costs, especially in middle-income countries. For all food groups other than fruits, the contributions of tariffs to diet costs declines in percentage terms as national per capita income increases.

## 6 Conclusions

This analysis provides new insights into the question of whether the least-cost items within recommended food groups are susceptible to costs associated with trade barriers. Matching retail items to importable commodities reveals that trade unit values account for about one-fifth of consumer prices. The remaining four-fifths of consumer prices are the cost of value-added services and other inputs needed for retail food provision. The role of domestic value added attenuates the impact of trade barriers on retail diet costs even for the minority of items that derive from imported commodities whose prices are raised by tariffs and non-tariff barriers. Almost half of least-cost items do not derive from an imported product, and those that do have relatively low tariff burdens. Trade barriers impose more meaningful costs in the case of least-cost vegetables and animal-source foods, particularly in South Asia and in Latin America and the Caribbean. The fact that South Asia would benefit more than other regions from tariff reduction is consistent with recent results from computable equilibrium (CGE) models, such as MIRAGRODEP. One analysis using this model finds that repurposing border measures including tariffs and NTMs has the most pronounced effect on the cost of healthy diets in South Asia (FAO, IFAD, UNICEF, WFP and WHO, 2022). However, the nature and extent of NTMs remain poorly understood as implemented by individual importers and as applied to distinct imported commodities. Nonetheless, NTMs appear to contribute more than tariffs to diet costs in the present analysis. Incorporating bilateral commodity-level AVEs of NTMs is an important area for future research that will allow for more nuanced estimates of the contribution of NTMs to the retail prices of least-cost items.

Our results can be seen as the trade-policy counterpart to recent findings that farmgate prices constitute a relatively small fraction of retail food spending even in low- and middle-income countries (Yi et al., 2021). That study used input–output data to explicitly account for the cost of labor, facilities and other resources used at each stage of food value chains in 61 countries, finding that farmgate prices received by growers averaged only 27% of retail costs paid by consumers. Our corresponding observation is that the trade unit value for imported bulk commodities that could be subject to import tariffs cost averaged 17% of retail item prices, and 22% among least-cost foods needed for healthy diets. Taken together, these two studies demonstrate the important difference between farmgate and wholesale markets for food commodities that drive agricultural income and employment, versus consumer markets for retail items that drive access to a healthy diet.

This study is subject to important limitations. Our analysis of trade barriers does not include import quotas or export barriers, which also play an important role in domestic and international price dynamics and could therefore influence the cost of healthy diets. Our analysis of the cost of compliance with NTMs is hampered by a lack of granular AVE estimates at the item level, and the work presented in this paper thus relies on GTAP sector-level estimates for global trade. Poor data coverage also limits the NTM analysis, particularly in Sub-Saharan Africa and East Asia and the Pacific where less than half of all economies were represented in 2017. Furthermore, trade data employed in this paper only reflect formal trade flows, which likely exclude a substantial share of actual trade, particularly in regions with extensive and porous borders. For example, under-reporting has been estimated to range from 11 to 40 percent in Sub-Saharan Africa (Mold and Chowdhury, 2021; Harding, 2019).

Finally, we note that any trade reform scenario would lead to change in prices and quantities. Predicting the magnitude of change would require specifying an equilibrium model of adjustment in supply and demand for each product, and possibly also considering the macroeconomic effects on employment, government revenue and exchange rates of any particular trade policy change. Those changes could be estimated with existing global and national models of agricultural production and trade. Our finding is that openness to trade is important for access to ingredients and intermediate inputs, while the value added in domestic food manufacturing, distribution and retailing accounts for most of the consumer prices that determine the cost and affordability of healthy diets.

In summary, availability of low-cost foods at the farmgate or port of entry is a necessary but not sufficient step for each population’s physical and economic access to a healthy diet. Beyond cost-reducing policies for commodities and bulk products, actions to make healthy diets affordable must address the entire value chain to the final consumer. Increased efficiency in distribution and retailing can help lower prices for the least-cost healthy diets identified in this analysis, and even when low-priced options are available those most at risk of malnutrition often have insufficient income to afford the quantities required for a healthy diet, and therefore need safety nets and nutrition assistance. Unhealthy foods may also enter to displace a healthy diet, calling for other interventions related to nutrition information, education, and regulation. Managing trade policy to ensure access to a diversity of products and origins is important for functioning food markets and promoting livelihoods in agriculture and the agribusiness sector, while research and policy on access to healthy diets can focus primarily on drivers of retail market conditions and other determinants of consumer choices.

## Funding

This study was commissioned by the Food and Agriculture Organization of the United Nations under PO351527, as part of the Food Prices for Nutrition project at Tufts University funded by the Bill & Melinda Gates Foundation and the Foreign, Commonwealth and Development Office of the UK as INV-016158.

Draft for submission to *World Development*, last revised 4 January 2024.

## CRediT authorship contribution statement

**Rachel Gilbert:** Data curation, Formal analysis, Visualization, Writing – original draft, Writing – review & editing. **Leah Costlow:** Data curation, Formal analysis, Visualization, Writing – original draft, Writing – review & editing. **Julia Matteson:** Funding acquisition, Project administration. **Jakob Rauschendorfer:** Conceptualization, Funding acquisition, Supervision, Writing – original draft, Investigation. **Ekaterina Krivonos:** Conceptualization, Funding acquisition, Investigation, Supervision, Writing – original draft. **Steven A. Block:** Methodology, Writing – original draft, Writing – review & editing. **William A. Masters:** Conceptualization, Formal analysis, Methodology, Supervision, Writing – original draft, Writing – review & editing.

## Declaration of competing interest

The authors declare that they have no known competing financial interests or personal relationships that could have appeared to influence the work reported in this paper.

## Data Availability

The authors do not have permission to share data.
