# Supplementary material for: Trade policy reform, retail food prices and access to healthy diets worldwide
Source: World Dev. 2024 May;177:106535. doi: 10.1016/j.worlddev.2024.106535 (PMC10958742; doi:10.1016/j.worlddev.2024.106535)
Supplement: Supplementary data 1 [file mmc1.docx]

**Supplementary Information**

This appendix contains information that is supplemental to “Trade policy reform, retail food prices and access to healthy diets worldwide.”

Contents

[Appendix 1 2](#_Toc155189008)

[Table A1. Least-cost diet baskets in three countries, with and without tariffs or NTMs 2](#_Toc155189009)

[Table A2. Sensitivity analysis for contribution of tariffs to CoHD by country 3](#_Toc155189010)

[Figure A1. Percentage range of diet costs attributable to tariffs, by region 7](#_Toc155189011)

[Figure A2. Sources and number of observations, data transformation, and results of the study 8](#_Toc155189012)

[Appendix 2 9](#_Toc155189013)

[Trade barriers: tariffs and non-tariff measures 10](#_Toc155189014)

[Measurement error, data cleaning, and summary of the analytical dataset 12](#_Toc155189015)

# Appendix 1

## Table A1. Least-cost diet baskets in three countries, with and without tariffs or NTMs

|  | **Least-cost diet with trade barriers** | |  | **Least-cost diet without tariffs** | |  | **Least-cost diet without non-tariff measures** | |
| --- | --- | --- | --- | --- | --- | --- | --- | --- |
|  |  |  |  |  |  |  |  |  |
|  | **ICP item name** | **Cost** |  | **ICP item name** | **Cost** |  | **ICP item name** | **Cost** |
| **Nigeria** | Maize grains | 0.34 |  | Maize grains | 0.34 |  | Maize grains | 0.33 |
|  | Broken rice | 0.41 |  | Broken rice | 0.41 |  | Broken rice | 0.41 |
|  | Fresh carrots | 0.23 |  | Fresh carrots | 0.21 |  | Fresh carrots | 0.21 |
|  | Fresh onions | 0.24 |  | Fresh onions | 0.24 |  | Fresh onions | 0.22 |
|  | Fresh cucumber | 0.47 |  | Fresh cucumber | 0.47 |  | Fresh cucumber | 0.47 |
|  | Banana | 0.24 |  | Banana | 0.22 |  | Banana | 0.22 |
|  | Large Mango (Grafted) | 0.28 |  | Large Mango (Grafted) | 0.28 |  | Large Mango (Grafted) | 0.28 |
|  | Milk | 0.42 |  | Milk | 0.37 |  | Milk | 0.35 |
|  | Beef Merguez | 0.49 |  | Beef Merguez | 0.49 |  | Beef Merguez | 0.49 |
|  | Spotted beans | 0.23 |  | Spotted beans | 0.23 |  | Spotted beans | 0.23 |
|  | Palm oil unrefined | 0.13 |  | Palm oil unrefined | 0.13 |  | *Peanut oil* | *0.12* |
|  |  | **3.49** |  |  | **3.40** |  |  | **3.34** |
|  |  |  |  |  |  |  |  |  |
|  | **ICP item name** | **Cost** |  | **ICP item name** | **Cost** |  | **ICP item name** | **Cost** |
| **Bangladesh** | Maize | 0.16 |  | Maize | 0.16 |  | Maize | 0.14 |
|  | Wholemeal flour | 0.20 |  | *Wholemeal flour* | *0.20* |  | Wholemeal flour | 0.20 |
|  | Fresh cabbage | 0.16 |  | Fresh carrots | 0.14 |  | Fresh carrots | 0.14 |
|  | Water spinach | 0.16 |  | Fresh cabbage | 0.16 |  | Fresh cabbage | 0.16 |
|  | Fresh carrots | 0.16 |  | Water spinach | 0.16 |  | Water spinach | 0.16 |
|  | Coconut | 0.21 |  | Coconut | 0.21 |  | Coconut | 0.21 |
|  | Fresh bananas | 0.37 |  | Fresh bananas | 0.37 |  | Fresh bananas | 0.37 |
|  | Chicken egg | 0.47 |  | Chicken egg | 0.47 |  | Chicken egg | 0.47 |
|  | Whole duck | 0.56 |  | Milk | 0.51 |  | Milk | 0.50 |
|  | Dhal | 0.23 |  | Dhal | 0.23 |  | Dhal | 0.20 |
|  | Palm oil | 0.12 |  | Palm oil | 0.11 |  | Palm oil | 0.11 |
|  |  | **2.79** |  |  | **2.70** |  |  | **2.65** |
|  |  |  |  |  |  |  |  |  |
|  | **ICP item name** | **Cost** |  | **ICP item name** | **Cost** |  | **ICP item name** | **Cost** |
| **Honduras** | Wheat flour | 0.29 |  | Wheat flour | 0.29 |  | Wheat flour | 0.29 |
|  | Long-grain rice | 0.35 |  | Long-grain rice | 0.34 |  | Long-grain rice | 0.35 |
|  | Fresh carrots | 0.17 |  | Fresh carrots | 0.17 |  | Fresh carrots | 0.17 |
|  | Fresh cabbage | 0.24 |  | Fresh cabbage | 0.24 |  | Fresh cabbage | 0.23 |
|  | Fresh onions | 0.26 |  | Fresh onions | 0.26 |  | Fresh onions | 0.26 |
|  | Fresh bananas | 0.16 |  | Fresh bananas | 0.16 |  | Fresh bananas | 0.16 |
|  | Fresh oranges | 0.32 |  | Fresh oranges | 0.32 |  | Fresh oranges | 0.32 |
|  | Milk | 0.48 |  | Milk | 0.48 |  | Milk | 0.48 |
|  | Chicken egg | 0.49 |  | Chicken egg | 0.49 |  | Chicken egg | 0.49 |
|  | Red Kidney beans | 0.23 |  | Red Kidney beans | 0.23 |  | Red Kidney beans | 0.21 |
|  | Vegetable oil | 0.25 |  | Vegetable oil | 0.25 |  | Vegetable oil | 0.25 |
|  |  | **3.25** |  |  | **3.23** |  |  | **3.22** |

Table A2. Sensitivity analysis for contribution of tariffs to CoHD by country

| Country | Baseline CoHD (2017) | Maximum AVE | Main result | Minimum AVE | Mean change in CoHD net of tariffs (%) | Standard deviation |
| --- | --- | --- | --- | --- | --- | --- |
| Albania | 4.00 | 3.95 | 3.96 | 3.99 | -0.96 | 0.55 |
| Algeria | 3.76 | 3.66 | 3.66 | 3.67 | -2.58 | 0.16 |
| Antigua and Barbuda | 4.11 | 3.97 | 3.97 | 4.06 | -2.79 | 1.22 |
| Argentina | 3.34 | 3.34 | 3.34 | 3.34 | 0.00 | 0.00 |
| Armenia | 3.10 | 3.08 | 3.08 | 3.09 | -0.32 | 0.19 |
| Australia | 2.14 | 2.14 | 2.14 | 2.14 | -0.02 | 0.01 |
| Austria | 2.62 | 2.61 | 2.61 | 2.61 | -0.41 | 0.09 |
| Azerbaijan | 2.35 | 2.34 | 2.34 | 2.35 | -0.23 | 0.16 |
| Bahamas, The | 4.12 | 4.04 | 4.04 | 4.10 | -1.61 | 0.83 |
| Bahrain | 3.38 | 3.36 | 3.36 | 3.38 | -0.47 | 0.37 |
| Bangladesh | 2.79 | 2.70 | 2.70 | 2.73 | -2.66 | 0.56 |
| Belarus | 3.17 | 3.16 | 3.17 | 3.17 | -0.19 | 0.15 |
| Belgium | 2.71 | 2.69 | 2.70 | 2.71 | -0.42 | 0.23 |
| Belize | 2.49 | 2.42 | 2.42 | 2.47 | -2.32 | 1.16 |
| Benin | 3.55 | 3.54 | 3.54 | 3.55 | -0.17 | 0.16 |
| Bhutan | 4.42 | 4.38 | 4.42 | 4.42 | -0.38 | 0.58 |
| Bolivia | 3.55 | 3.54 | 3.54 | 3.55 | -0.15 | 0.13 |
| Bosnia and Herzegovina | 3.93 | 3.93 | 3.93 | 3.93 | -0.05 | 0.05 |
| Botswana | 3.54 | 3.54 | 3.54 | 3.54 | 0.00 | 0.00 |
| Brazil | 2.81 | 2.79 | 2.81 | 2.81 | -0.23 | 0.29 |
| Brunei Darussalam | 4.00 | 4.00 | 4.00 | 4.00 | 0.00 | 0.00 |
| Bulgaria | 3.86 | 3.85 | 3.85 | 3.85 | -0.29 | 0.10 |
| Burkina Faso | 3.20 | 3.20 | 3.20 | 3.20 | -0.11 | 0.09 |
| Burundi | 2.99 | 2.90 | 2.97 | 2.99 | -1.19 | 1.60 |
| Cabo Verde | 3.41 | 3.37 | 3.37 | 3.39 | -0.95 | 0.41 |
| Cambodia | 3.52 | 3.20 | 3.52 | 3.52 | -3.11 | 5.26 |
| Cameroon | 2.74 | 2.74 | 2.74 | 2.74 | 0.00 | 0.00 |
| Canada | 2.72 | 2.72 | 2.72 | 2.72 | -0.01 | 0.00 |
| Central African Republic | 3.42 | 3.37 | 3.37 | 3.42 | -0.99 | 0.85 |
| Chile | 2.95 | 2.95 | 2.95 | 2.95 | -0.01 | 0.02 |
| China | 2.52 | 2.51 | 2.51 | 2.52 | -0.27 | 0.04 |
| Colombia | 2.96 | 2.94 | 2.94 | 2.96 | -0.34 | 0.29 |
| Congo, Dem. Rep. | 2.89 | 2.89 | 2.89 | 2.89 | 0.00 | 0.00 |
| Congo, Rep. | 3.34 | 3.30 | 3.30 | 3.32 | -1.10 | 0.30 |
| Costa Rica | 3.84 | 3.80 | 3.80 | 3.84 | -0.58 | 0.50 |
| Cote d'Ivoire | 3.30 | 3.27 | 3.28 | 3.29 | -0.43 | 0.25 |
| Croatia | 4.23 | 4.23 | 4.23 | 4.23 | -0.11 | 0.07 |
| Cyprus | 2.87 | 2.86 | 2.87 | 2.87 | -0.15 | 0.22 |
| Czech Republic | 2.96 | 2.95 | 2.95 | 2.95 | -0.21 | 0.05 |
| Denmark | 2.35 | 2.34 | 2.34 | 2.34 | -0.52 | 0.14 |
| Djibouti | 2.80 | 2.80 | 2.80 | 2.80 | 0.00 | 0.00 |
| Dominica | 4.00 | 3.98 | 3.98 | 4.00 | -0.34 | 0.30 |
| Ecuador | 2.79 | 2.78 | 2.78 | 2.79 | -0.25 | 0.22 |
| Egypt, Arab Rep. | 3.57 | 3.56 | 3.56 | 3.57 | -0.19 | 0.16 |
| Estonia | 3.61 | 3.59 | 3.59 | 3.60 | -0.52 | 0.19 |
| Eswatini | 3.43 | 3.43 | 3.43 | 3.43 | -0.02 | 0.03 |
| Ethiopia | 3.24 | 3.11 | 3.11 | 3.23 | -2.96 | 2.20 |
| Fiji | 3.61 | 3.49 | 3.49 | 3.58 | -2.53 | 1.53 |
| Finland | 2.61 | 2.59 | 2.59 | 2.60 | -0.62 | 0.14 |
| France | 2.83 | 2.81 | 2.82 | 2.82 | -0.49 | 0.21 |
| Gabon | 3.41 | 3.28 | 3.29 | 3.41 | -2.42 | 2.09 |
| Gambia, The | 3.05 | 3.05 | 3.05 | 3.05 | 0.00 | 0.00 |
| Germany | 2.64 | 2.63 | 2.63 | 2.63 | -0.36 | 0.03 |
| Ghana | 3.75 | 3.60 | 3.60 | 3.75 | -2.70 | 2.29 |
| Greece | 3.06 | 3.06 | 3.06 | 3.06 | -0.09 | 0.07 |
| Grenada | 5.33 | 5.26 | 5.26 | 5.33 | -0.96 | 0.84 |
| Guinea | 3.77 | 3.77 | 3.77 | 3.77 | 0.00 | 0.00 |
| Guyana | 4.63 | 4.44 | 4.57 | 4.60 | -1.98 | 1.87 |
| Honduras | 3.25 | 3.23 | 3.23 | 3.25 | -0.49 | 0.42 |
| Hong Kong SAR, China | 3.56 | 3.56 | 3.56 | 3.56 | 0.00 | 0.00 |
| Hungary | 3.43 | 3.42 | 3.42 | 3.42 | -0.07 | 0.04 |
| Iceland | 2.67 | 2.66 | 2.66 | 2.67 | -0.20 | 0.18 |
| India | 2.80 | 2.75 | 2.75 | 2.79 | -1.15 | 0.89 |
| Indonesia | 4.07 | 4.06 | 4.06 | 4.07 | -0.04 | 0.03 |
| Iran, Islamic Rep. | 3.01 | 3.01 | 3.01 | 3.01 | 0.00 | 0.00 |
| Ireland | 2.28 | 2.26 | 2.26 | 2.26 | -0.73 | 0.17 |
| Israel | 2.36 | 2.29 | 2.30 | 2.36 | -1.94 | 1.69 |
| Italy | 2.83 | 2.82 | 2.83 | 2.83 | -0.23 | 0.06 |
| Jamaica | 6.34 | 6.25 | 6.25 | 6.34 | -0.98 | 0.83 |
| Japan | 5.55 | 5.52 | 5.52 | 5.55 | -0.35 | 0.31 |
| Jordan | 3.32 | 3.31 | 3.31 | 3.32 | -0.23 | 0.20 |
| Kazakhstan | 2.39 | 2.39 | 2.39 | 2.39 | -0.05 | 0.03 |
| Kenya | 2.97 | 2.81 | 2.91 | 2.97 | -2.38 | 2.74 |
| Korea, Rep. | 4.92 | 4.77 | 4.79 | 4.90 | -2.00 | 1.40 |
| Kuwait | 3.28 | 3.27 | 3.28 | 3.28 | -0.17 | 0.17 |
| Kyrgyz Republic | 2.97 | 2.97 | 2.97 | 2.97 | 0.00 | 0.00 |
| Latvia | 3.17 | 3.17 | 3.17 | 3.17 | -0.07 | 0.05 |
| Lithuania | 3.05 | 3.04 | 3.04 | 3.04 | -0.22 | 0.06 |
| Luxembourg | 2.31 | 2.27 | 2.27 | 2.28 | -1.67 | 0.31 |
| Madagascar | 3.12 | 3.11 | 3.12 | 3.12 | -0.13 | 0.11 |
| Malawi | 2.74 | 2.74 | 2.74 | 2.74 | -0.11 | 0.08 |
| Malaysia | 3.12 | 3.09 | 3.09 | 3.12 | -0.52 | 0.45 |
| Maldives | 3.49 | 3.49 | 3.49 | 3.49 | 0.00 | 0.00 |
| Mali | 3.03 | 2.94 | 2.94 | 3.01 | -2.16 | 1.30 |
| Malta | 3.60 | 3.56 | 3.59 | 3.60 | -0.44 | 0.48 |
| Mauritania | 3.45 | 3.43 | 3.43 | 3.45 | -0.38 | 0.20 |
| Mauritius | 3.34 | 3.34 | 3.34 | 3.34 | 0.00 | 0.00 |
| Mexico | 3.04 | 3.04 | 3.04 | 3.04 | -0.09 | 0.14 |
| Moldova | 2.46 | 2.44 | 2.45 | 2.46 | -0.39 | 0.30 |
| Mongolia | 4.61 | 4.59 | 4.59 | 4.60 | -0.41 | 0.17 |
| Montenegro | 3.46 | 3.40 | 3.43 | 3.46 | -0.83 | 0.86 |
| Morocco | 2.71 | 2.30 | 2.69 | 2.70 | -5.29 | 8.47 |
| Namibia | 3.26 | 3.25 | 3.25 | 3.26 | -0.04 | 0.04 |
| Nepal | 3.95 | 3.89 | 3.89 | 3.94 | -1.00 | 0.75 |
| Netherlands | 2.69 | 2.68 | 2.68 | 2.68 | -0.32 | 0.05 |
| New Zealand | 2.53 | 2.53 | 2.53 | 2.53 | 0.00 | 0.00 |
| Nicaragua | 3.39 | 3.38 | 3.38 | 3.39 | -0.04 | 0.03 |
| Niger | 2.84 | 2.83 | 2.83 | 2.84 | -0.29 | 0.23 |
| Nigeria | 3.49 | 3.40 | 3.40 | 3.44 | -2.13 | 0.60 |
| North Macedonia | 3.37 | 3.34 | 3.35 | 3.37 | -0.52 | 0.46 |
| Norway | 3.37 | 3.33 | 3.37 | 3.37 | -0.36 | 0.62 |
| Oman | 2.81 | 2.81 | 2.81 | 2.81 | -0.06 | 0.05 |
| Pakistan | 3.40 | 3.39 | 3.39 | 3.40 | -0.27 | 0.19 |
| Panama | 4.17 | 4.04 | 4.04 | 4.17 | -2.05 | 1.77 |
| Paraguay | 3.43 | 3.43 | 3.43 | 3.43 | -0.09 | 0.08 |
| Peru | 2.97 | 2.97 | 2.97 | 2.97 | -0.03 | 0.03 |
| Philippines | 3.86 | 3.84 | 3.84 | 3.86 | -0.28 | 0.23 |
| Poland | 2.96 | 2.96 | 2.96 | 2.96 | -0.01 | 0.01 |
| Portugal | 2.55 | 2.54 | 2.55 | 2.55 | -0.15 | 0.10 |
| Qatar | 2.39 | 2.39 | 2.39 | 2.39 | -0.22 | 0.08 |
| Romania | 3.03 | 3.02 | 3.02 | 3.03 | -0.28 | 0.12 |
| Russian Federation | 3.17 | 3.16 | 3.16 | 3.17 | -0.18 | 0.16 |
| Rwanda | 2.82 | 2.71 | 2.82 | 2.82 | -1.32 | 2.28 |
| Sao Tome and Principe | 3.30 | 3.26 | 3.27 | 3.30 | -0.65 | 0.58 |
| Saudi Arabia | 3.44 | 3.43 | 3.43 | 3.44 | -0.14 | 0.07 |
| Senegal | 2.30 | 2.26 | 2.26 | 2.29 | -1.03 | 0.78 |
| Serbia | 4.18 | 4.18 | 4.18 | 4.18 | 0.00 | 0.00 |
| Seychelles | 3.97 | 3.96 | 3.96 | 3.97 | -0.35 | 0.23 |
| Sierra Leone | 2.93 | 2.93 | 2.93 | 2.93 | 0.00 | 0.00 |
| Singapore | 2.77 | 2.77 | 2.77 | 2.77 | 0.00 | 0.00 |
| Slovak Republic | 3.22 | 3.21 | 3.21 | 3.22 | -0.15 | 0.11 |
| Slovenia | 2.84 | 2.83 | 2.84 | 2.84 | -0.15 | 0.11 |
| South Africa | 4.07 | 4.06 | 4.06 | 4.07 | -0.07 | 0.06 |
| Spain | 2.50 | 2.49 | 2.49 | 2.49 | -0.28 | 0.07 |
| Sri Lanka | 3.58 | 3.54 | 3.54 | 3.58 | -0.62 | 0.53 |
| St. Kitts and Nevis | 3.00 | 2.95 | 2.95 | 3.00 | -1.14 | 0.99 |
| St. Lucia | 3.33 | 3.21 | 3.21 | 3.33 | -2.44 | 1.97 |
| St. Vincent and the Grenadines | 4.13 | 3.98 | 3.99 | 4.13 | -2.37 | 1.94 |
| Suriname | 4.85 | 4.85 | 4.85 | 4.85 | 0.00 | 0.00 |
| Sweden | 3.17 | 3.16 | 3.16 | 3.17 | -0.29 | 0.08 |
| Switzerland | 2.45 | 2.45 | 2.45 | 2.45 | 0.00 | 0.00 |
| Taiwan | 3.92 | 3.90 | 3.91 | 3.91 | -0.20 | 0.13 |
| Tanzania | 2.68 | 2.66 | 2.67 | 2.68 | -0.36 | 0.40 |
| Thailand | 3.98 | 3.98 | 3.98 | 3.98 | 0.00 | 0.00 |
| Trinidad and Tobago | 3.93 | 3.93 | 3.93 | 3.93 | 0.00 | 0.00 |
| Tunisia | 3.35 | 3.32 | 3.32 | 3.35 | -0.58 | 0.41 |
| Turkey | 2.98 | 2.97 | 2.97 | 2.97 | -0.13 | 0.08 |
| Uganda | 2.85 | 2.85 | 2.85 | 2.85 | 0.00 | 0.00 |
| United Arab Emirates | 2.75 | 2.75 | 2.75 | 2.75 | -0.19 | 0.11 |
| United Kingdom | 1.67 | 1.65 | 1.65 | 1.66 | -0.66 | 0.22 |
| United States | 3.20 | 3.20 | 3.20 | 3.20 | -0.01 | 0.02 |
| Uruguay | 3.07 | 3.06 | 3.06 | 3.07 | -0.26 | 0.22 |
| Zambia | 3.09 | 3.09 | 3.09 | 3.09 | -0.08 | 0.07 |
| Zimbabwe | 3.49 | 3.48 | 3.48 | 3.49 | -0.23 | 0.20 |
|  |  |  |  |  |  |  |

***Note***: “Maximum AVE” denotes the contribution of tariffs to diet costs when traded commodities are matched to tariff lines subject to the largest possible tariff out of the pool of potential matches. “Minimum AVE” denotes the scenario where commodities are matched to the lowest possible tariff. “Main result” denotes the primary analysis scenario, in which the best possible match is manually chosen as the least-processed version from the pool of potential matches.

## Figure A1. Percentage range of diet costs attributable to tariffs, by region

***Note***: “Maximum AVE” denotes the contribution of tariffs to diet costs when traded commodities are matched to tariff lines subject to the largest possible tariff out of the pool of potential matches. “Minimum AVE” denotes the scenario where commodities are matched to the lowest possible tariff. “Primary Match” denotes the primary analysis scenario, in which the best possible match is manually chosen as the least-processed version from the pool of potential matches.

## Figure A2. Sources and number of observations, data transformation, and results of the study


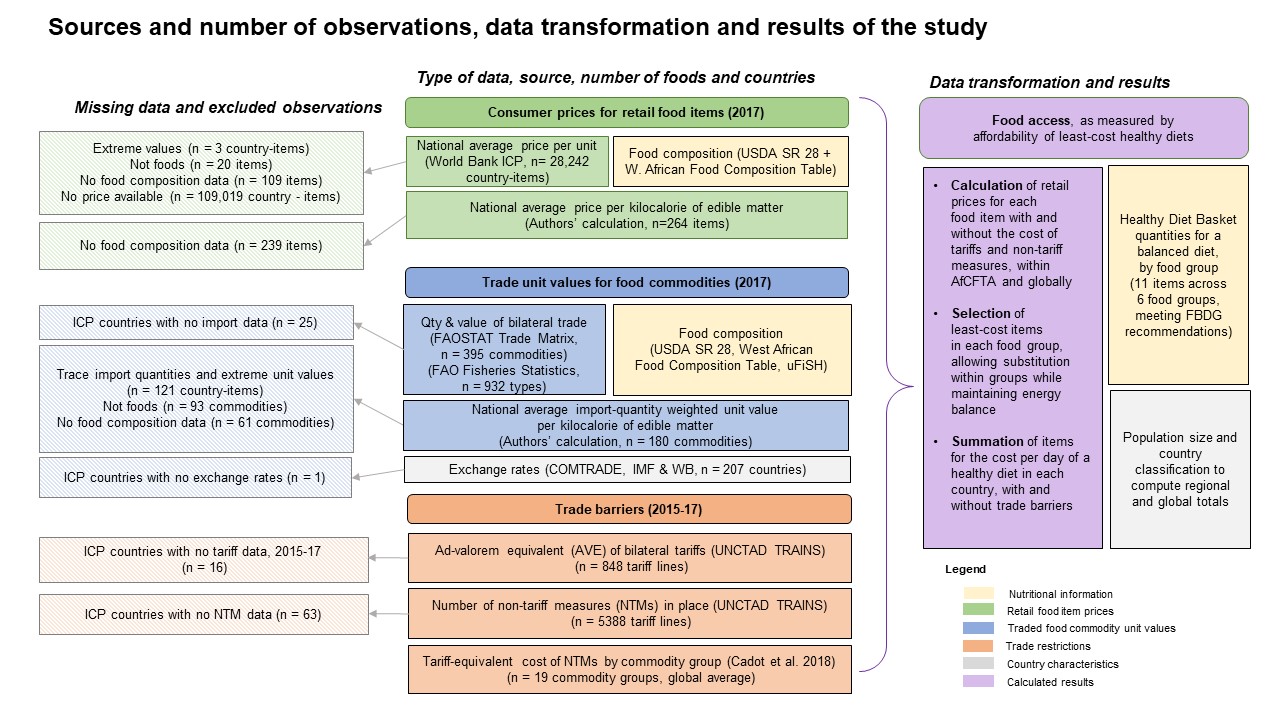


# Appendix 2

**Import unit values**: To estimate the cost of the traded commodities that correspond to each retail item, we calculate import unit values from the FAOSTAT Detailed Trade Matrix data as the annual value of imports divided by the annual import quantity for each FAO traded food commodity. Import unit values are calculated for 263 traded food commodity categories (FAO, 2022a). FAOSTAT's trade matrix does not include fish and seafood products, so fish and seafood import unit values are derived from import values and quantities from FAO's Fisheries and Aquaculture database on global fish trade (FAO, 2022c). For agricultural commodities, we estimate a national weighted-average import unit value for each country, using import quantities at the bilateral level as weights to correspond with available tariff data, which use import value weights. As FAO does not publish bilateral trade quantities for fish and seafood, fish and seafood import unit values are unweighted.

Import values are downloaded from FAOSTAT in 2017 USD and converted to 2017 PPP for comparability with the ICP retail food prices. We convert import values to 2017 local currency units using exchange rates from COMTRADE and then to 2017 PPP using the ICP 2017 PPP for household consumption expenditure. COMTRADE exchange rates are those used to convert national trade data as reported to the UN into 2017 USD. For countries without a COMTRADE exchange rate we convert the reported currency to local currency units using exchange rates from the IMF International Financial Statistics or the World Bank before final conversion to 2017 PPP terms.

Import unit values are converted into kilocalories of edible matter using food composition data from the United States Department of Agriculture (USDA), the West African Food Composition Table, and the FAO/INFOODS Global food composition database for fish and shellfish (uFiSh1.0) (FAO, 2016).

## Trade barriers: tariffs and non-tariff measures

We draw data from various sources to capture the magnitude and extent of tariffs and NTMs. We use tariff data from the TRAINS database, which represents the most exhaustive and up-to-date database. Bilateral tariff data are published as ad valorem equivalents (AVEs) at the 6-digit Harmonised System (HS) level through the WITS platform, where data are available for 193 reporting countries, with tariff years from 1988 through 2020. The data covers all traded commodities under the harmonized system (HS) nomenclature. For each tariff line, we use 2017 tariffs and keep the most recent data available for each country in 2015 or 2016 for countries without 2017 tariff data. All trade barriers reported in earlier HS nomenclatures are converted to the HS 2017 nomenclature. After matching to imported FAO foods, this results in 2015 tariffs for 173 HS6 codes, 2016 tariffs for 199 HS6 codes, and 2017 tariffs for 218 HS6 codes.

We use the weighted average of tariffs imposed by the importing country on all trading partners for imported food commodities. This weighted average is calculated as the total duty levied divided by the total value of imports, across all trading partners from which the importing country receives the specified food commodity under consideration. Import values are obtained from TRAINS at the HS6 level and may or may not reflect trade data from the same calendar year as the available tariff data. All tariffs are downloaded as AVEs and converted into specific tariffs, expressed as USD per edible kilocalorie of the product.

We build on an existing one-to-many concordance between the FAO Commodity List (FCL) and the Harmonized System nomenclature downloaded from FAOSTAT (FAO, 2022b), using ICP and FAOSTAT metadata to choose the best match. For example, when matching the ICP-FCL pair “Beef without bones” (ICP) and “Meat, cattle, boneless” (FCL) to an HS code, both HS code 20130 “Meat; of bovine animals, boneless cuts, fresh or chilled” and 20230 “Meat; of bovine animals, boneless cuts, frozen” are reasonable matches. In these scenarios, we matched to the rawest form of the product, in this case “fresh or chilled” rather than frozen.

We calculate the retail price contribution of NTMs using AVEs estimated at the global GTAP-sector level by Cadot *et al.* (2018), also expressed in the form of cost per edible kilocalorie of each product. Cadot et al. (2018) include SPS, TBT, border control measures (BCM) and quantitative restrictions (QRs). To apply these estimates to retail food items, their matched FAO traded food commodities were manually matched to the corresponding GTAP sector. For any HS6 product group that has a non-zero number of NTMs, we apply the complete AVE estimate for the corresponding GTAP sector. While various publicly available databases estimate AVEs of NTMs at a more disaggregated level, none include data for 2017 or adjacent years or for countries in sub-Saharan Africa (Sanjuán López *et al.*, 2021). These authors estimate the trade effects of NTMs for roughly 5,000 traded goods across 80 countries.

## Measurement error, data cleaning, and summary of the analytical dataset

We identified outliers in the TRAINS tariff data by identifying those tariffs that lie more than 150% of the IQR above the 75^th^ percentile for each traded commodity, flagging 360 tariffs at the HS6 level imposed by 79 importing countries. We reviewed each tariff and cross-checked with tariff data published in the [Market Access Map](https://www.macmap.org/) (MACMAP) to confirm whether the tariff appeared to be correct as published in TRAINS. This process was limited by the inherently complex nature of tariff implementation. Tariffs downloaded from TRAINS reflect most favoured nation status and schedules, preferential trade agreements, tariff rate quotas, and other types of trade policies, which are exceedingly difficult to track even in their raw form. We therefore took a cautious approach and only revised tariffs in cases where an error was clearly present in the TRAINS database. We only replaced the TRAINS data with MACMAP data if the TRAINS weighted average AVE tariff was larger than the MACMAP most favoured nation rate, as the complexity of trade agreements could result in a smaller but almost never a larger average weighted average rate. We also replaced TRAINS data when MACMAP included tariff data from a more recent year. For 341 of the flagged tariffs, the MACMAP database closely corresponded to the value downloaded from TRAINS, and therefore we retained these tariffs in our analysis. For the remaining 19 flagged tariffs, we recalculated the global weighted average AVE tariff using the bilateral tariff data available through MACMAP. For 3 of these tariff lines, the data available through MACMAP were more recent than those available through TRAINS. In the case of two extraordinarily large Indonesian tariffs, we replaced the TRAINS data with MACMAP data from 2013.

The ICP dataset on retail prices in 2017 is provided by the World Bank in its near-final form, after extensive averaging for use in purchasing power parity calculations. After matching to food composition data, of the 28,242 item prices we found only three instances where the reported price per calorie was implausibly low, perhaps due to reporting errors in the reported unit of measure of item description. In contrast, the bilateral import unit values we derived from the FAOSTAT trade matrix had very wide variance, perhaps due to misreporting of quantities or prices. Of the 37,260 potential national average trade unit values (180 commodities imported into 207 countries), the trade matrix reports import quantities and values for a total of 8,403 product-country flows. Of those, we exclude 898 observations that we thereby reclassify as non-traded commodities for that country, because the trade unit values were outliers for their commodity using the 1.5*IQR criterion, and imported in trace quantities under 5,000 MT/year by that country. We then winsorised the remaining trade unit values at the 25^th^ and 75^th^ percentile of observations for each commodity, to allow variance around the median without the full range of extreme values in the raw data. Even so, 134 of the resulting trade unit values exceeded the retail price and were excluded, reclassifying the retail item as nontraded due to lack of suitable match to a traded commodity.
